# Embryonic ethanol exposure alters expression of *sox2* and other early transcripts in zebrafish, producing gastrulation defects

**DOI:** 10.1038/s41598-020-59043-x

**Published:** 2020-03-03

**Authors:** Swapnalee Sarmah, Rajneesh Srivastava, Jeanette N. McClintick, Sarath C. Janga, Howard J. Edenberg, James A. Marrs

**Affiliations:** 1 Department of Biology, Indiana University-Purdue University Indianapolis, Indianapolis, IN 46202 USA; 2 Department of BioHealth Informatics, Indiana University-Purdue University Indianapolis, Indianapolis, IN 46202 USA; 3 Department of Biochemistry and Molecular Biology, Indiana University School of Medicine, Indianapolis, IN 46202 USA

**Keywords:** Gastrulation, Embryology

## Abstract

Ethanol exposure during prenatal development causes fetal alcohol spectrum disorder (FASD), the most frequent preventable birth defect and neurodevelopmental disability syndrome. The molecular targets of ethanol toxicity during development are poorly understood. Developmental stages surrounding gastrulation are very sensitive to ethanol exposure. To understand the effects of ethanol on early transcripts during embryogenesis, we treated zebrafish embryos with ethanol during pre-gastrulation period and examined the transcripts by Affymetrix GeneChip microarray before gastrulation. We identified 521 significantly dysregulated genes, including 61 transcription factors in ethanol-exposed embryos. Sox2, the key regulator of pluripotency and early development was significantly reduced. Functional annotation analysis showed enrichment in transcription regulation, embryonic axes patterning, and signaling pathways, including Wnt, Notch and retinoic acid. We identified all potential genomic targets of 25 dysregulated transcription factors and compared their interactions with the ethanol-dysregulated genes. This analysis predicted that Sox2 targeted a large number of ethanol-dysregulated genes. A gene regulatory network analysis showed that many of the dysregulated genes are targeted by multiple transcription factors. Injection of *sox2* mRNA partially rescued ethanol-induced gene expression, epiboly and gastrulation defects. Additional studies of this ethanol dysregulated network may identify therapeutic targets that coordinately regulate early development.

## Introduction

Fetal alcohol spectrum disorder (FASD) is caused by the exposure to ethanol during prenatal developmental^1–3^. FASD patients display a range of morphological deformities and neurological deficits, including characteristic craniofacial dysmorphology, cognitive impairment, sensory defects, motor disabilities and organ deformities. A recent meta-analysis of FASD among children and youth showed the prevalence is approximately 0.8% globally, but it exceeds 1% in 76 countries^4^. The World Health Organization (WHO) European Region has the highest prevalence of FASD (1.98%) followed by the WHO Region of Americas (0.88%)^4^. Among all the countries studied to date, FASD is the most prevalent in South Africa where the prevalence is as high as 11.1%^4^. FASD prevalence is notably higher among special populations, for example, low socioeconomic status populations^5,6^, children in orphanages, people in psychiatric care etc.^4^.

Despite various proposed mechanisms to explain FASD etiology, the molecular targets of ethanol toxicity during development are poorly understood. Conception through gastrulation are sensitive periods for ethanol-induced defects^7,8^. During this period stem and progenitor cells transition from pluripotency to one of the three germ layers, and the cells undergo coordinated movements to organize the body plan^9,10^. These effects are regulated transcriptionally, for example, through the maternal to zygotic transition and the pluripotency transcriptional circuit. Since mammalian embryos develop inside their mother, it is difficult to study the effects of ethanol during gastrulation.

The zebrafish is an outstanding model to study early stages of embryogenesis because zebrafish produce hundreds of embryos synchronized at the same developmental stage and the embryos develop outside their mother^9,11^. There are morphological differences in early development stages between fish and humans. However, developmental gene expression networks are highly conserved from fish to human^12^. Zebrafish is an established model for the study of embryonic ethanol-exposure effects on development and its functional consequences, providing insights into the potential mechanisms of ethanol teratogenicity^13–15^.

Ethanol treatment of zebrafish embryos from 2 to 24 hours post-fertilization (hpf) reproducibly causes robust FASD-like defects, including craniofacial, cardiac, and neural defects^14–21^. This is a model for chronic ethanol exposure during early stages of pregnancy, when mothers may not know they are pregnant and may continue to drink alcohol. Our previous studies provided evidence of critical signaling dysregulation during organogenesis, including BMP, Notch, Wnt, and retinoic acid, which leads to heart and eye defects^18,20^. Phenotypic differences between ethanol-treated and untreated embryos were first detected during gastrulation, when ethanol-treated embryos displayed reduced epiboly progression^22–24^. Our studies examining changes in gene expression in ethanol-exposed embryos at mid-gastrulation (8 hpf) using microarray gene expression analysis identified various dysregulated genes including cell adhesion molecule Protocadherin-18a^22^. Defects in epiboly and gastrulation cell movements in ethanol-exposed embryos resembled the phenotype of embryos deficient in cadherin cell adhesion^25^. Protocadherin-18a was reduced after ethanol exposure, and injection of mRNA encoding protocadherin-18a partially rescued epiboly progression, cellular morphology of the enveloping layer cells during gastrulation, and convergence-extension of the anterior-posterior axes^22^. However, the ethanol dysregulated genes identified during mid-gastrulation could include indirect effects of ethanol exposure. We hypothesize that developmental signaling pathway defects seen during morphogenesis and organogenesis represent pleiotropic effects of ethanol on gene expression patterns that begin at the earliest stages of embryogenesis, when the embryo is first exposed to ethanol.

Gastrulation is a highly sensitive period for ethanol-induced defects^7,8^. In humans, gastrulation occurs at implantation and is the first time of exposure to maternal blood circulation and, hence, to maternal blood alcohol. Initial development is controlled by the maternally deposited proteins and mRNAs, until a burst of zygotic genes are transcribed in the early embryo prior to gastrulation that ultimately controls development^26,27^. In zebrafish, gastrulation starts around 5.25 hpf when the blastomere cells cover 50% of the yolk cells^10,28^. The initial zygotic transcriptional burst occurs ahead of gastrulation, during midblastula transition around 3 hpf^27^. The pluripotency factors Nanog, Pou5f1 (Oct4) and SoxB1, activate the zygotic program and pre-gastrulation development in zebrafish^27^. Zebrafish SoxB1 comprises of six *sox* genes: *sox1a/1b/2/3/19a/19b*^29^, of which, *sox19b* is supplied maternally^30^. These maternal factors play fundamental roles in activating transcription during early embryogenesis. Most of the initially activated genes guide early development^27^. The gene encoding the transcription factor Sox2 is one of the earliest zygotic genes activated (around 30% epiboly or 4.3 hpf)^30^. Sox2 plays critical roles in early vertebrate development by maintaining pluripotency and promoting differentiation later. This study examined the effects of ethanol on early transcripts before gastrulation. Affymetrix GeneChip microarray was performed and ethanol dysregulated genes that include critical transcription factors were identified. A gene regulatory network involving transcription factors and their target genes was identified. Experiments revealed significant reduction in *sox2* transcripts and dysregulation of Sox2 target genes after ethanol exposure. Injecting *sox2* mRNA partially rescued ethanol defects in early zebrafish embryos, showing an important role in FASD genesis.

## Results

### Ethanol exposure during blastula period perturbs gene expression patterns prior to gastrulation

**Figure 1:**
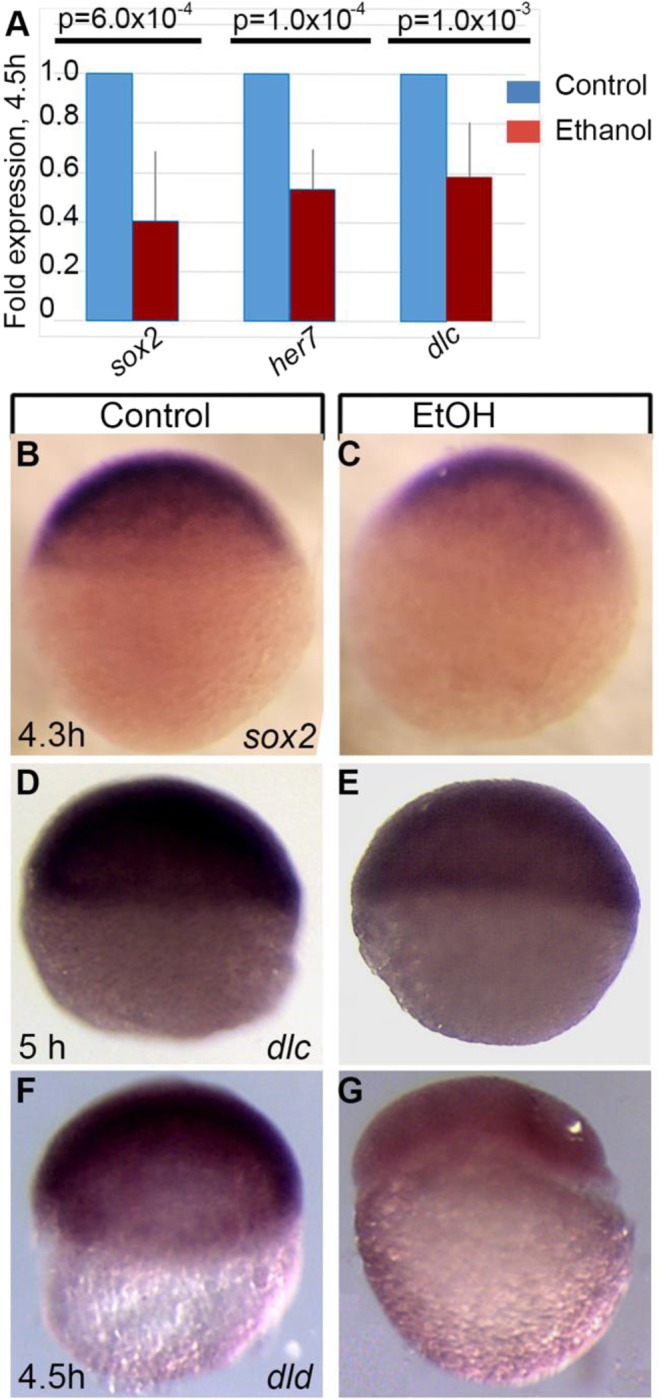
Ethanol exposure during mid-blastula transition changes the gene expression in zebrafish embryos. (**A**) Quantitative RT-PCR assays comparing transcript levels of *sox2, her7*, and *dlc* after ethanol treatment. Average fold change in expression was calculated from at least 3 independent experiments, with samples analyzed in triplicate. Samples were normalized to transcript levels for *rsp15*, and fold change for ethanol treated embryos was calculated by normalizing control levels to 1. (**B**–**G**) Whole mount *in situ* hybridization showed reduced expression of *sox2* (**B**,**C)**, *dlc* (**D**,**E**) and *dld* (**F**,**G**) in E100 embryos (**C**,**E**,**G**) compared to control (**B**,**D**,**F**).

Affymetrix GeneChip microarray analysis comparing control embryos to those treated with ethanol from 2 to 4.5 hpf (cleavage and pre-gastrulation stages) showed statistically significant changes of expression of many genes critical for embryogenesis (Supplementary Table S1) including *sox* genes, Notch ligands and *Hairy/E(spl)-related* (*her*) genes. To validate microarray results, a subset of genes were examined by either qPCR or by *in situ* hybridization. Downregulation of *sox2* (array fold change −1.99, p < 0.0001), *dlc* (array fold change −1.82, p = 0.007) and *her7* (array fold change −2.51, p < 0.001) genes at 4.5 hpf after ethanol exposure was confirmed by qPCR (Fig. 1A). *In situ* hybridization showed reduced staining for *sox2*, *dlc* and *dld* (array fold change −1.53, p < 0.001) at 4.5 hpf in ethanol-exposed embryos compared to control embryos (Fig. 1B–G).

There were significant changes in the expression of 651 probes, (absolute changes ≥ 1.25, FDR 0.15, p < 0.03) due to ethanol exposure (Supplementary Table S1). Out of those 651 probes, we were able to map Ensembl IDs for 534 probes, which correspond to 521 genes. Functional annotation analysis of ethanol dysregulated genes was done using DAVID that identified genes enriched in cellular processes, including transcription regulation and gene expression; DNA recombination; cell division and microtubule-based movement; cell-cell adhesion; and carbohydrate metabolic processes. Genes enriched in developmental processes, including dorso-ventral and anterior-posterior axes formation, cerebellum, somite, and optic fissure development were also detected in DAVID analysis. Dysregulated genes were enriched in Wnt, Notch, and retinoic acid signaling pathways (Table 1).

**Table 1:** Gene ontology analysis of ethanol-dysregulated genes.

Category	Term	Count	p-value
GOTERM_BP_DIRECT	GO:0006355~regulation of transcription, DNA-templated	45	0.016
GOTERM_BP_DIRECT	GO:0001756~somitogenesis	9	0.001
GOTERM_BP_DIRECT	GO:0005975~carbohydrate metabolic process	9	0.062
GOTERM_BP_DIRECT	GO:0031101~fin regeneration	8	0.001
GOTERM_BP_DIRECT	GO:0009953~dorsal/ventral pattern formation	7	0.050
GOTERM_BP_DIRECT	GO:0016055~Wnt signaling pathway	7	0.088
GOTERM_BP_DIRECT	GO:0007219~Notch signaling pathway	6	0.006
GOTERM_BP_DIRECT	GO:0009952~anterior/posterior pattern specification	6	0.007
GOTERM_BP_DIRECT	GO:0007018~microtubule-based movement	6	0.042
GOTERM_BP_DIRECT	GO:0000278~mitotic cell cycle	6	0.046
GOTERM_BP_DIRECT	GO:0001889~liver development	6	0.059
GOTERM_BP_DIRECT	GO:0021549~cerebellum development	5	0.001
GOTERM_BP_DIRECT	GO:0016337~single organismal cell-cell adhesion	5	0.030
KEGG_PATHWAY	dre00561:Glycerolipid metabolism	5	0.066
KEGG_PATHWAY	dre03320:PPAR signaling pathway	5	0.094
GOTERM_BP_DIRECT	GO:0048384~retinoic acid receptor signaling pathway	4	0.004
GOTERM_BP_DIRECT	GO:0001878~response to yeast	4	0.019
GOTERM_BP_DIRECT	GO:0018279~protein N-linked glycosylation via asparagine	4	0.041
GOTERM_BP_DIRECT	GO:0006310~DNA recombination	4	0.063
GOTERM_BP_DIRECT	GO:0001757~somite specification	3	0.038
GOTERM_BP_DIRECT	GO:0061386~closure of optic fissure	3	0.043
GOTERM_BP_DIRECT	GO:0009948~anterior/posterior axis specification	3	0.062
GOTERM_BP_DIRECT	GO:0001574~ganglioside biosynthetic process	3	0.076
GOTERM_BP_DIRECT	GO:0006094~gluconeogenesis	3	0.076
GOTERM_BP_DIRECT	GO:0036342~post-anal tail morphogenesis	3	0.098
GOTERM_BP_DIRECT	GO:0071539~protein localization to centrosome	2	0.070
GOTERM_BP_DIRECT	GO:0045814~negative regulation of gene expression, epigenetic	2	0.071
GOTERM_BP_DIRECT	GO:0061056~sclerotome development	2	0.093
REACTOME_PATHWAY	Polyamine metabolic process:R-DRE-351202:R-DRE-351202	2	0.099

Category refers to the original database where the terms orient; term refers to the enriched term associated to the gene list; count refers to the total number of differentially expressed genes annotated to a given gene ontology term; the smaller the p-value, the more enriched the term.

**Figure 2:**
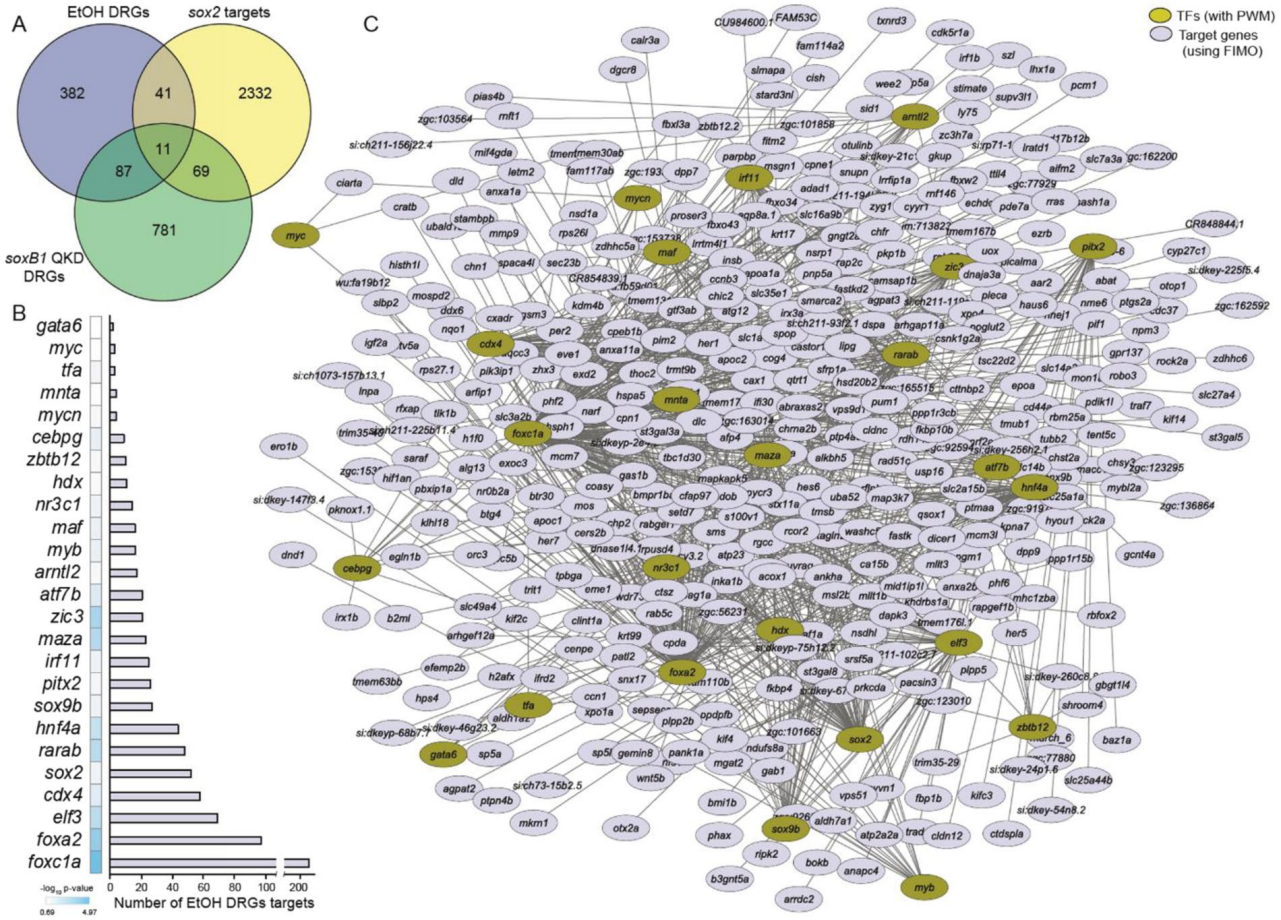
Ethanol induced gene expression changes during zygotic genome activation. (**A**) Venn diagram shows overlapping of ethanol dysregulated genes identified in the Affymetrix GeneChip microarray analysis of control and ethanol treated embryos at 4.5 hpf with potential Sox2 targets and the genes differentially expressed in quadruple knockdown of SoxB1 factors, which includes Sox2 (*Soxb1* QKD targets). (**B**) Twenty five of the ethanol-dysregulated transcription factors showing the enrichment of their targets in our ethanol-dysregulated gene set. (**C**) Transcription factor-target gene network visualizing using cytoscape shows interactions among the transcription factors (gold circles) and their target gene (blue circles) in our dataset. Ethanol dysregulated targets of Sox2 are co-shared by other dysregulated transcription factors.

Among the 521 dysregulated genes, we identified 61 transcription factors (Table 2), including Sox2, a critical transcription factor. The expression of *sox2* was significantly reduced after ethanol exposure. To identify Sox2 targets across the zebrafish genome, position weight matrixes for Sox2 were mapped within 2 kb upstream of transcription start sites of genes using find individual motif occurrences software^31^. Possible Sox2 targets were compared with the ethanol-dysregulated genes, which showed that 52 genes were common in both datasets (Fig. 2A). Transcriptome changes caused by SoxB1 knockdown (quadruple knockdown: *sox2/3/19a/19b*) at 30% epiboly (~4.7 hpf) were reported previously^29^. Results of this study were compared with ethanol dysregulated genes (4.5 hpf). We found 98 genes common in between SoxB1 knockdown dysregulated genes and ethanol dysregulated genes. Comparison of all three datasets showed 11 common genes in all these datasets (Fig. 2A). These data indicate that ethanol affects the expression of Sox2 and several Sox2 transcriptional targets.

**Table 2:** Ethanol-dysregulated transcription factors.

Gene Symbol	Fold change	p-value	PWM
*tfa*	−2.05	0.0016	Available
*sox2*	−1.99	0.0001	Available
*elf3*	−1.87	0.0003	Available
*si:ch211-222e23.7*	−1.78	0.0032	Available
*cdx4*	−1.62	0.0048	Available
*pitx2*	−1.62	0.0284	Available
*maf*	−1.47	0.0015	Available
*foxc1a*	−1.43	0.0002	Available
*zic3*	−1.32	0.0063	Available
*foxa2*	−1.31	0.0132	Available
*hnf4a*	−1.30	0.0155	Available
*gata6*	−1.26	0.0052	Available
LOC797948	1.28	0.0222	Available
*atf7b*	1.27	0.0082	Available
*mycn*	1.30	0.0100	Available
*arntl2*	1.32	0.0022	Available
*rarab*	1.36	0.0101	Available
*mycb*	1.37	0.0076	Available
*cebpg*	1.41	0.0082	Available
wu:fb82f02	1.46	0.0070	Available
*maza*	1.50	0.0219	Available
*myb*	1.57	0.0003	Available
*nr3c1*	1.70	0.0053	Available
*sox9b*	1.72	0.0005	Available
*irf11*	1.84	0.0030	Available
*her7*	−2.93	0.0003	Not Available
*msgn1*	−2.35	0.0012	Not Available
*her1*	−2.10	0.0126	Not Available
zgc:136639	−1.83	0.0026	Not Available
*sp5l*	−1.82	0.0014	Not Available
*otx1a*	−1.69	0.0138	Not Available
*irx1b*	−1.56	0.0193	Not Available
*irx3a*	−1.49	0.0119	Not Available
*nr0b2a*	−1.46	0.0011	Not Available
*eve1*	−1.41	0.0004	Not Available
*pknox1.1*	−1.39	0.0005	Not Available
*hes6*	−1.35	0.0062	Not Available
*lhx1a*	−1.34	0.0123	Not Available
*msxb*	−1.34	0.0091	Not Available
*nr0b2a*	−1.32	0.0045	Not Available
*sp5*	−1.27	0.0186	Not Available
LOC407678	1.26	0.0141	Not Available
*etv5a*	1.26	0.0235	Not Available
zgc:162349	1.27	0.0002	Not Available
zgc:165515	1.27	0.0058	Not Available
zgc:162349	1.27	0.0009	Not Available
*zorba*	1.28	0.0237	Not Available
*zhx3*	1.30	0.0050	Not Available
si:rp71-1g18.1	1.30	0.0134	Not Available
*znf277*	1.30	0.0185	Not Available
*lrrfip1a*	1.33	0.0045	Not Available
si:ch211-119o8.6	1.33	0.0086	Not Available
*lrrfip2*	1.35	0.0099	Not Available
*her5*	1.36	0.0023	Not Available
*rcor2*	1.37	0.0075	Not Available
*pias4l*	1.38	0.0259	Not Available
*terf1*	1.46	0.0108	Not Available
*klf2a*	1.46	0.0034	Not Available
LOC100149164	1.48	0.0037	Not Available
*etv5a*	1.48	0.0261	Not Available
si:dkeyp-68b7.7	1.50	0.0046	Not Available
LOC797322	1.66	0.0283	Not Available
*tsc22d2*	1.96	0.0028	Not Available
zgc:77060	2.51	0.0109	Not Available

To predict the possible binding sites of other ethanol-dysregulated transcription factors across the zebrafish genome, we explored the available position weight matrixes (TRANSFAC). Position weight matrixes were found for 24 of the 60 other ethanol-dysregulated transcription factors. Target genes of these 24 transcription factors were predicted by mapping position weight matrixes within 2 kb upstream of the start site of genes across zebrafish genome. Predicted targets of the dysregulated transcription factors show that many ethanol-dysregulated genes are targets of these transcription factors. We compared the predicted targets of these dysregulated transcription factors and examined for the enrichment of ethanol dysregulated genes by computing hypergeometric probability. This transcription factor-target gene interaction analysis identified 827 interactions that include 25 transcription factors targeting 423 dysregulated genes. Individual interaction counts for each transcription factor is listed in Table 3. The enrichment of ethanol-dysregulated targets over all possible genomic targets for a given transcription factor plotted as a bar graph is shown in Fig. 2B. A network analysis was done using cytoscape software to visualize the interactions between the dysregulated transcription factors and the dysregulated targets. This analysis showed that 25 transcription factors target many of the same ethanol dysregulated genes, which identifies a potential ethanol-induced transcription factor-target gene regulatory network in the early embryo (Fig. 2C). As many of the genes dysregulated by ethanol exposure were targeted by multiple dysregulated transcription factors, these factors could produce synergistic ethanol dysregulation effects during early zebrafish development.

**Table 3:** Ethanol-dysregulated transcription factors and the enrichment of their targets in the ethanol-dysregulated gene set.

Transcription factors	Potential targets in the ethanol-dysregulated dataset	p-value	−log_10_ p-value
*foxc1a*	207	0.0000	4.9689
*foxa2*	97	0.0001	3.8315
*zic3*	21	0.0006	3.2427
*maza*	23	0.0011	2.9524
*rarab*	48	0.0016	2.7962
*elf3*	69	0.0019	2.7307
*hnf4a*	44	0.0034	2.4676
*atf7b*	21	0.0153	1.8144
*cdx4*	58	0.0234	1.6299
*cebpg*	9	0.0312	1.5052
*myb*	16	0.0391	1.4078
*maf*	16	0.0454	1.3426
*irf11*	25	0.0537	1.2701
*sox9b*	27	0.0540	1.2679
*nr3c1*	14	0.0546	1.2631
*sox2*	52	0.0567	1.2465
*tfa*	3	0.0739	1.1315
*pitx2*	26	0.0793	1.1008
*arntl2*	17	0.0952	1.0215
*mycn*	4	0.1035	0.9852
*zbtb12*	10	0.1105	0.9568
*hdx*	11	0.1218	0.9143
*mnta*	4	0.1541	0.8121

### Injection of *sox2* mRNA partially rescues ethanol-induced epiboly, gastrulation and gene expression defects

**Figure 3:**
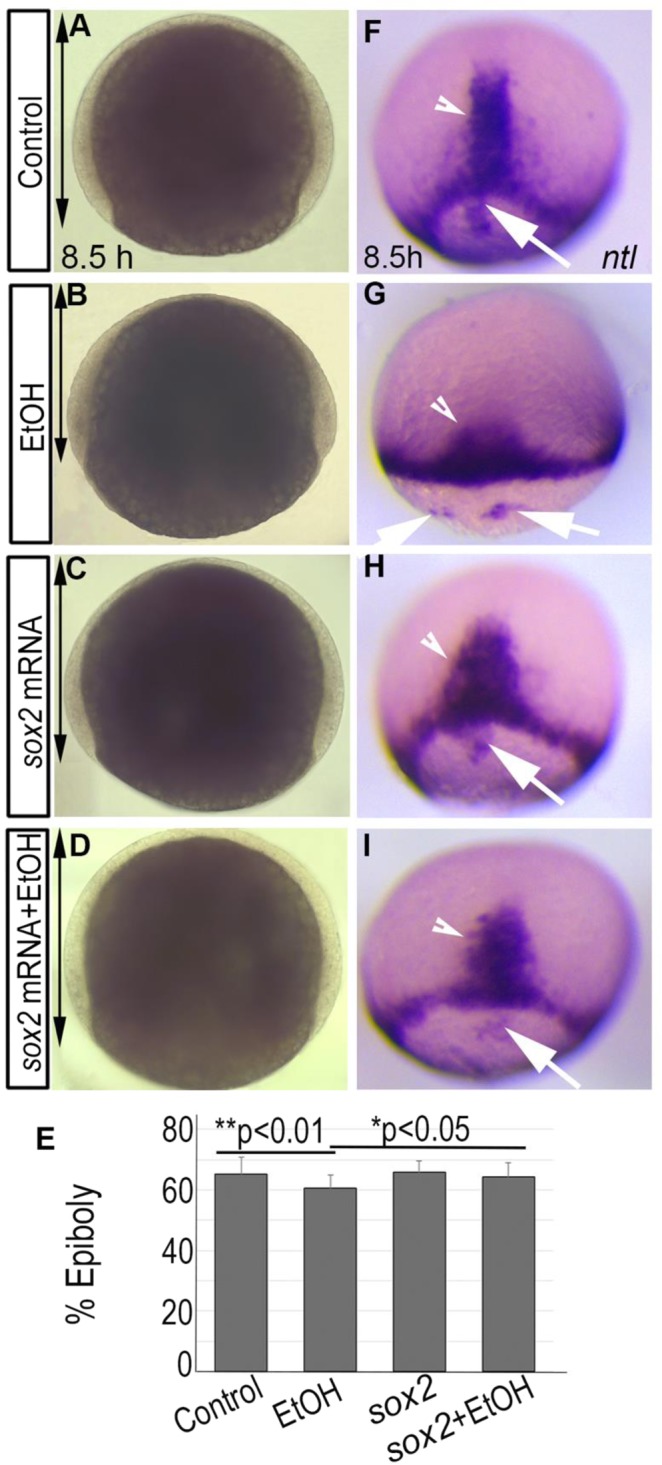
Ethanol induced epiboly and gastrulation defect was partially rescued by *sox2* mRNA injection. (**A**–**D**) Bright field images showed reduced epiboly progression after ethanol exposure (B; double arrow), which was rescued by *sox2* mRNA injection (**D**). (**E**) Graph shows the percentage of epiboly progression in control, ethanol-treated, *sox2* mRNA injected, and *sox2* mRNA + ethanol-treated injected embryos (see text for statistics). (**F**–**I**) *In situ* hybridization detecting *ntl* expression shows dorsal forerunner cells closely associated to the germ band in control, embryo (**F**), *sox2* mRNA injected (**H**), and ethanol-treated + *sox2* mRNA injected (**I**) embryos, and a dramatic separation and fragmentation of the dorsal forerunner cells from the germ band and from each other in the ethanol treated embryo (**G**).

Sox2 plays a critical role in pluiripotency and embryogenesis^32,33^. The pluripotency transcriptional circuit, which includes Sox2, regulates pre-gastrulation events in the zebrafish embryos^27,29^. Thus, Sox2 was selected for additional studies. To test the role of Sox2 activity in ethanol toxicity, 2–4 cell stage embryos were injected with 25 pg of synthetic *sox2* mRNA to determine whether restoring this gene function remedies ethanol-induced defects. Epiboly progression was measured in the injected embryos after ethanol exposure and compared with controls. Untreated embryos reached 65.30 ± 5.5% epiboly at 8 hpf, but ethanol-treated embryos only reached 60.68 ± 4.2%, a significant delay (control *vs*. ethanol-treated, p < 0.05) (Fig. 3A,B,E). Average epiboly progression of *sox2* mRNA injected embryos without ethanol treatment was 65.80 ± 3.6% at 8 hpf (Fig. 3C,E). Injection of *sox2* mRNA with ethanol treatment rescued epiboly delay (64.39 ± 4.4%; control *vs. sox2* mRNA + ethanol-treated, p = 0.72, ethanol-treated *vs*. sox2 mRNA + ethanol-treated, p < 0.05) (Fig. 3A–E).

To mark the axial mesendoderm, germ band (mesodermal cells at the leading-edge during epiboly progression) and the dorsal forerunner cells (a group of cells that migrate at the leading-edge of shield during gastrulation but do not involute) in control and treated embryos, *ntl in situ* hybridization was performed. Control embryos had dorsal forerunner cells closely associated with the germ ring. Dorsal forerunner cells were dissociated from one another and from the germ band in ethanol-treated embryos, which was partially rescued by *sox2* mRNA injection (Fig. 3F–I). Functional annotation analysis detected dysregulation of genes involved in dorsal/ventral and anterior/posterior axes formation (Table 1). *ntl* staining confirmed that ethanol-exposed embryos had reduced convergence-extension of the axial mesendoderm cells compared to control embryos, producing shorter and wider axes. The convergence-extension defect was partially rescued by *sox2* mRNA injection in the ethanol treated embryos (Fig. 3F–I).

**Figure 4:**
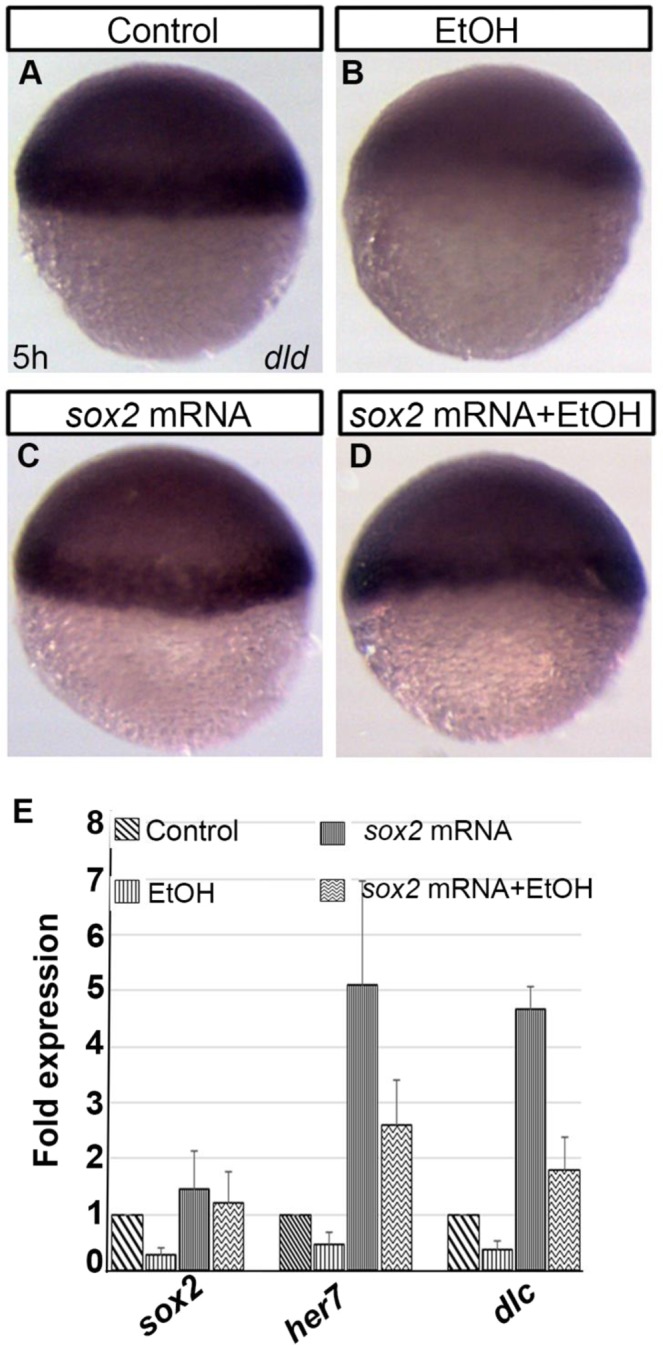
Ethanol induced gene expression changes was reversed by *sox2* mRNA injection. (**A**–**D**) Whole mount ISH shows reduced *dld* expression after ethanol exposure, which was like control in sox2 mRNA injected and sox2 mRNA injected plus ethanol-treated embryos. (**E**) Quantitative PCR showed downregulation of *sox2, her7*, and *dlc* transcripts in the ethanol-treated embryos, which increased in the sox2 mRNA injected plus ethanol-treated embryos.

The effects of *sox2* mRNA injection on the expression of a few ethanol-dysregulated genes were analyzed. The expression level of *dld* was restored in *sox2* mRNA injected, ethanol-exposed embryos as seen by *dld in situ* hybridization (Fig. 4A–D). Quantitative PCR was done to analyze the expression of *sox2, her7*, and *dlc*. The *sox2* mRNA injection restored expression of *sox2* that was downregulated in ethanol-treated embryos (Fig. 4E). The reduced expression of *her7* and *dlc* in ethanol-treated embryos was significantly restored by *sox2* mRNA injection in ethanol-treated embryos (ethanol-treated *vs*. sox2 mRNA + ethanol-treated: *dlc*; p < 0.01, *her7*; p < 0.01) (Fig. 4E). However, the expression levels of these genes in *sox2* mRNA injected + ethanol-treated embryos were higher compared to control embryos (control *vs*. sox2 mRNA injected + ethanol-treated: *dlc*; p < 0.01, *her7*; p < 0.01) (Fig. 4E). Overall, *sox2* mRNA injection partially rescued ethanol-induced early developmental defects.

## Discussion

Mouse and rat studies showed the association between prenatal ethanol exposure and gene expression changes in postnatal and adult stages^34–37^. This study examined ethanol-induced gene expression changes during embryogenesis before gastrulation. This is the first animal model study that identified the effects of ethanol on a master regulator, Sox2 that orchestrates embryogenesis, self-renewal, and pluripotency^27,29,32^. Ethanol exposure altered the expression of a large number of genes, which include other critical regulators of development. The differentially expressed genes are involved in various functions ranging from cellular processes, embryonic development to signaling pathways. This indicates possible multifactorial effects, which may include the alteration of epigenome by ethanol exposure, causing the changes in expression of critical genes.

Functional annotation analysis of the dysregulated genes identified enrichment of genes involved in mitotic nuclear division and microtubule-based movements. In fact, our previous study identified large multinucleated enveloping layer cells in 8 hpf ethanol-treated embryos and fragmentation of yolk microtubules^18^, which support current findings. The cell adhesion defects that was observed previously at 8 hpf^18^ were detected at 4.5 hpf ethanol-treated embryos, suggesting continuous defects in cell-to-cell communication and cell movements in those embryos. Interestingly, ethanol-sensitive signaling pathways detected during early embryogenesis were Wnt, Notch, and retinoic acid. Ethanol-induced dysregulation of retinoic acid signaling pathway was reported earlier^16,17,19,38–41^. Our studies examining the heart and the eye in ethanol-treated embryos identified disruption of Wnt, Notch, retinoic acid and Bmp signaling pathways during organogenesis^17–20^. This study showed that those signaling defects initiate early in ethanol-exposed embryos and continue to have their detrimental effects at later stages of development.

This microarray analysis detected a reduction of *sox2* expression. Ethanol-induced effects on stoichiometry of Sox2 and Oct4 was reported during differentiation of mouse embryonic stem cell^42^. Sox2 and other Sox B1 type transcriptional regulators control a wide range of developmental effectors, including *pcdh18a* (gastrulation movement), *neurog1*, *hesx1*, and *zic1* (neural differentiation), *oep*, and *shh* (neural patterning)^29^. Our previous Affymetrix GeneChip microarray study (GEO accession: GSE48380) comparing genes in control and ethanol-exposed embryos during mid-gastrulation identified significant dysregulation of all these genes after ethanol exposure at 8 hpf^15,22^. Reduction of Sox2 prior to gastrulation might be responsible for the dysregulation of these genes, interfering with gastrulation and other developmental events. Notably, convergence-extension defects seen in the ethanol-exposed embryos was also reported in the SoxB1 knockdown embryos, which display similar wedge-shaped *ntl* expression pattern. Presence of precise amount of *sox2* transcripts is essential for normal development of the embryo. Injection of more than 30 pg of *sox2* mRNA into the embryos caused delayed epiboly progression and other developmental defects, which caused the death of injected embryos (data not shown). Injection of 25 pg of *sox2* mRNA partially rescued ethanol-induced epiboly defects. Injection of less than 25 pg of *sox2* mRNA gave weaker rescue effects. *sox2* mRNA injection raised the *her7* and *dlc* transcript levels in ethanol-exposed embryos. However, the *her7* and *dlc* transcript levels in *sox2* mRNA-injected + ethanol-exposed embryos were significantly higher than the transcript levels in control embryos. Although *sox2* mRNA injection did not fully rescue *her7* and *dlc* expression in ethanol-exposed embryos, the results support their transcriptional regulation by Sox2 and ethanol. The transcription factor-target gene regulatory network (Fig. 2C) shows that *her7* is regulated not only by Sox2, but also by Mnt, Cebpg3, and Atf7. Similarly, the expression of *dlc* is regulated by Sox2, Elf3, and Maz.

Transcription factor-target gene interaction analysis showed that the number of potential targets among the ethanol-induced dysregulated genes are more for Foxc1a and Foxa2 (members of forkhead transcription factors), Elf3 (a member of the E26 transformation specific family of transcription factors), and Cdx4 (a member of caudal gene family) than for Sox2. However, ethanol-induced gene expression change for *sox2* was higher (and possibly playing a more significant role) than *foxc1a*, *foxa2*, *elf3* and *cdx4*. Foxc1a plays crucial role in somitogenesis, cardiovascular and retina development during embryogenesis^43–48^. Foxa transcription factors are essential for developmental of dorsal axis structures including prechordal plate, notochord, hypochord, and floor plate^49–51^. A growing body of research shows that Elf3 plays significant roles in the development of cancer^52–56^. Although poorly understood, there is evidence that Elf3 is important during development. A null mutation of Elf3 caused the death of about 30% of mice *in utero*^57,58^. Another Sox family gene *sox9b* was also detected as the ethanol-dysregulated gene in our study. Sox9 is involved in many developmental processes including craniofacial, heart, brain and retinal development in mammal^59^. Zebrafish has two homologues of *sox9*, *sox9a* and *sox9b*. Both Sox9a and Sox9b play roles on neural, cardiac, and cartilage development Zebrafish^60–64^. Network analysis showed that many of these dysregulated transcription factors interact with each other, which suggests that the ethanol-induced developmental defects were the combined and perhaps synergistic effects of multiple regulators and that manipulation of one gene would not lead to complete rescue of ethanol-induced defects. Future study is needed to analyze the potential interaction between combinations of genes coordinately disrupt early development, contributing to ethanol-induced defects.

## Methods

### Zebrafish husbandry

Zebrafish (*Danio rerio*) ABTL strain was raised and maintained under standard laboratory conditions^65^ following Indiana University Policy on Animal Care and Use. The use of zebrafish adults for breeding, embryo collection and embryo experiments were approved by the campus animal care and use ethics committee: IUPUI School of Science Institutional Animal Care and Use Committee (IACUC).

### Ethanol treatment

Embryos were kept in embryo medium after fertilization. At 2 hpf, embryos were divided into two groups. One group was transferred to embryo medium containing 100 mM ethanol (E100) and the other group was kept in embryo medium without ethanol (control).

### RNA isolation, microarray analysis, and bioinformatics approach

Ethanol-treated and untreated embryos were incubated until 4.5 hpf. At this point, total RNA was extracted from pools of 20 control and 20 E100 embryos using TRIzol reagent (Sigma, St Louis, MO, USA). Seven independent experiments were done. RNA samples were purified by passing through the Qiagen RNAeasy column (Cat. No. 74104). The RNA quality was examined by Agilent Bioanalyzer RNA Nanochip (Agilent Technologies, Santa Clara, CA, USA). The RNA integrity number (RIN) for one of the ethanol samples was 2.5, so that experiment (both treated and control) was not further analyzed. For the remaining 12 samples (from 6 experiments) RIN ≥ 8.2. Standard protocol for the Affymetrix 3′IVT Express kit (Affymetrix, Santa Clara, CA, USA) was followed to label the samples starting with 100 ng of total RNA. The 12 samples were each hybridized to a Zebrafish Genome Array (Affymetrix) for 17 h, washed, stained and scanned following the standard protocol; all 12 were handled in parallel as a single batch. Arrays were visually scanned for abnormalities or defects; none were found.

Affymetrix gene expression console software was used to generate MAS5^66^ signals and detection calls; arrays were scaled to a target of 1000. Only those probe sets that had a MAS5 signal fraction present ≥ 0.50 in at least one of the two treatments were analyzed^67^. MAS5 signals were imported into Partek Genomics Suite (Partek, Inc., St Louis, MO, USA) and log_2_ transformed. These log_2_ transformed signals were used for principal components analysis, hierarchical clustering and signal histograms to determine if there were any outlier arrays, and no outliers were detected. A 2-way ANOVA with factors for treatment (ethanol *vs*., control) and independent experiment (random effect) was used to analyze log_2_ transformed signals. This analysis indicated that the embryo batch was indeed significant. The False Discovery Rate (FDR) was calculated using the Storey qvalue method^68^. Microarray data were deposited in the NCBI GEO database, accession number GSE145574.

A subset of the differentially expressed probes with absolute changes ≥ 1.25 were included in downstream analysis (FDR 0.15; p < 0.03). We performed function annotation analysis of these ethanol dysregulated probes using DAVID^69^. Additionally, we annotated the transcription factors for these differentially expressed probe IDs using AnimalTFDB database^70^. For these annotated ethanol-dysregulated transcription factors, we obtained the position weight matrices available in TRANSFAC^71^ and searched for their occurrence using “find individual motif occurrences” algorithm^31^ to predict the target site within 2 kb upstream of start site of every gene in zebrafish genome (Zv11). Find individual motif occurrences computes a log-likelihood ratio score for the occurrence of each motif in a specific input sequence and hence enables the discovery of recognition sequences of transcription factors in the upstream regions of gene starts. Find individual motif occurrences computes converts these scores into p-values using dynamic programming generated at random genomic loci with user defined background frequencies for genomic alphabet (A, T, G, C) with false discovery rates. It provides a ranked list of motif occurrences per position weight matrices, each with an associated log-likelihood ratio score, p-value and other statistical metrics. Find individual motif occurrences computes based predicted targets of these dysregulated transcription factors were compared and examined for the enrichment of ethanol induced dysregulated targets by computing hypergeometric probability for the gene set enrichment. Resulting data was represented as networks using cytoscape^72^ network visualization software.

### Quantitative PCR analysis

One µg of total RNA extracted from control and E100 embryos was reverse transcribed to cDNA using M-MLV reverse transcriptase (Promega, Madison, WI, USA), and cDNA was diluted tenfold with RNase free water. Each 20 µl PCR reaction was performed with 1–4 µl of cDNA using Power SYBR Green PCR mix (Applied Biosystems/Life Technologies, Inc.) and 0.5 µM of each primer. Primer sets used are listed in the Supplementary Table S2. Three independent experiments were performed on the 7300 Real Time PCR System (Applied Biosystems), each in triplicate, using *rsp15* as internal control. Relative expression was calculated as described^73^. Fold changes in gene expression was calculated using comparative C_T_ method (ΔΔC_T_)^73^. Unpaired two-tailed student’s *t*-test was used for comparisons between control and ethanol treated groups using GraphPad software (GraphPad Software, La Jolla, CA, USA).

### *In situ* hybridization

Whole-mount *in situ* hybridization of zebrafish embryos was performed using digoxigenin-labeled riboprobes for *ntl, dlc*, and *dld*. The riboprobes were synthesized using DIG RNA Labeling Kit (Roche, Indianapolis, IN, USA) according to manufacturer’s recommendations. For *sox2* riboprobe, pCBA3-zf-sox2 plasmid was cut using BamHI restriction enzyme, and the Dig RNA probe was synthesized using T7 RNA polymerase. Images were collected using a Leica MZ12 microscope equipped with Leica DFC290 camera.

### *sox2* rescue experiments

mRNA was synthesized from a pCBA3-zf-sox2 vector^29^ using a SP6 mMessage mMachine kit (Ambion, Austin, TX, USA). Synthetic mRNA (25 pg/embryo) was injected into the embryos at the 2-cell stage. Injected and uninjected embryos were treated with or without 100 mM ethanol until analyzed. For epiboly measurement, embryos were fixed at 8 hpf, dechorionated and imaged focusing on enveloping cell layer at the embryo margin. Percent epiboly progression was calculated using Image J software. For gen expression analyses, embryos were dechorionated and total RNA was extracted at 4.3 hpf, and quantitative PCR was performed. One-way ANOVA and post hoc Tukey HSD for individual comparisons were used for analyses in rescue experiments.

## Supplementary information

Supplementary Table S1

Supplementary Table S2
