# Supplementary material for: Embryonic ethanol exposure alters expression of sox2 and other early transcripts in zebrafish, producing gastrulation defects
Source: Sci Rep. 2020 Mar 3;10:3951. doi: 10.1038/s41598-020-59043-x (PMC7054311; doi:10.1038/s41598-020-59043-x)
Supplement: Supplementary file 2 — Supplementary Table S2 [file 41598_2020_59043_MOESM2_ESM.pdf]

Supplemental Information for:
Embryonic ethanol exposure alters expression of *sox2* and other early transcripts in zebrafish, producing gastrulation defects

Swapnalee Sarmah, Rajneesh Srivastava, Jeanette N. McClintick, Sarath C. Janga, Howard J. Edenberg, James A. Marrs

Supplementary Table 1: List of differentially expressed probes.

See Excel spreadsheet.

Supplementary Table 2: Primer used are quantitative PCR reactions.

**Primer name**	**Sequences**
sox2-F	GGGCACGGGGAACACCAACT
sox2-R	TGGTCGCTTCTCGCTCTCGG
her7-F	AGGTCACTCCAGAAAGCAGCACGC
her7-R	TGGCGGGAGGTTGGTTGAGC
dlc-F	CGCTCAGATCCACCTCTGTAGACACA
dlc-R	TGTTCGGGAATGATGTACACAGGATG
rsp15-F	CAGAGGTGTGGACCTGGACCAGC
rsp15-R	CGGGCAGGATGACCATGTCTCTC
